# Methodological considerations in assessing HPV prevalence in Barrett’s oesophagus progression

**DOI:** 10.1038/s41416-025-03168-1

**Published:** 2025-09-19

**Authors:** Talita H. A. de Oliveira, Lesley A. Anderson, Stephanie G. Craig, Helen G. Coleman, Tarik Gheit, Sandrine McKay-Chopin, Jacqueline Jamison, Damian T. McManus, Christopher R. Cardwell, Victoria Bingham, Brian T. Johnston, Jacqueline A. James, Andrew T. Kunzmann

**Affiliations:** 1 Patrick G. Johnston Centre for Cancer Research, Queen’s University Belfast, Belfast, Antrim UK; 2 Aberdeen Centre for Health Data Science, University of Aberdeen, Aberdeen, UK; 3 Centre for Public Health, Queen’s University Belfast, Belfast, Antrim UK; 4 International Agency for Research on Cancer-World Health Organization, Lyon, France; 5 Northern Health and Social Care Trust, Belfast, Antrim UK; 6 Institute of Pathology, Queen’s University Belfast, Belfast, Antrim UK; 7 Precision Medicine Centre, Queen’s University Belfast, Belfast, Antrim UK; 8 Belfast Health and Social Care Trust, Belfast, Antrim UK; 9 Northern Ireland Biobank, Queen’s University Belfast, Belfast, Antrim UK

**Keywords:** Oesophageal cancer, Cancer genetics

We read with interest the correspondence by Rajendra and Rabiei regarding our recent study on human papillomavirus (HPV) prevalence in Barrett’s oesophagus (BO) progression to oesophageal adenocarcinoma (OAC) [1]. The authors raise important concerns about our nested case-control study, particularly regarding the absence of dysplastic samples, the potential for false negatives, and the absence of p16 immunohistochemistry.

The low prevalence of HPV in BO progressor samples and negative results in cancer samples prevent us from using precious finite dysplastic samples, and still don’t explain the lack of findings in the cancer samples. The previous study referenced by Rajendra and Rabiei investigated cross-sectional comparisons of different sets of BO, dysplasia and OAC patients; therefore, conclusions about the transition between disease progression cannot be made [2].

We share their concerns that DNA/RNA degradation in older FFPE samples (1993–2013) could impact sensitivity. However, we expect the magnitude of this misclassification to be modest. A cervical cancer study found consistent viral DNA detection in samples taken 15 years previously and only noted a minor drop off in sensitivity in samples stored for up to 85 years [3]. Furthermore, previous population-based studies of oropharyngeal cancer from the same Biobank as our study have reliably detected HPV DNA within FFPE tissue, with only a modest drop in sensitivity with sample age [4]. Additionally, the sensitivity of the multiplex assay has been demonstrated in multiple studies, and the ability to detect multiple strains of HPV, whilst our use of β-globin amplification to ensure DNA integrity does not guarantee sensitivity for HPV detection, it does reduce the risk of considerable deficits. Therefore, large sensitivity deficits seem unlikely to fully explain differences with the small proportion of studies identifying high HPV prevalence. In addition, as we mentioned in our research article, the β-globin gene was amplified in each of the multiplex assays, and only samples positive for β-globin amplification were included in the analysis.

As a research team, our greater concern was preventing cross-contamination and reducing the likelihood of false positive results. In our previous systematic review of HPV prevalence in oesophageal adenocarcinoma, concerns over cross-contamination were a key issue [5]. Indeed, we implemented a pilot phase using negative external controls, which revealed that even small discrepancies from procedures lead to false positives. Specificity concerns also guided our decision to use RNA ISH instead of relying on p16 staining, since up to nearly 30% of oropharyngeal cancer cases showed discordant results of p16 positive and HPV negative [6]. Keeping in mind the criteria suggested by Dr Magnus von Knebel Döberitz about the pipeline of analysis needed to stablish a causal role for HPV in non-anogenital cancers, if HPV was not detected in cancer samples, the result is sufficient to determine that there is no causal role association, without the need to investigate a surrogate maker such as p16 [7]. Our RNA ISH analysis went even further than just normal HPV detection (i.e., qPCR for HPV L1 or L2 amplification), increasing the detection method specificity and sensitivity by focusing on HPV16 and HPV18 E6/E7 mRNA [4].

The lab that performed the RNA ISH experiments for our study has extensive experience in running the commercial RNAScope assay on an automated platform across a range of tissues, including the HPV assay for 18 high-risk genotypes on oropharyngeal tissues, which demonstrated high sensitivity for detection (89%, 95% CI: 81–95) [4, 8]. This study utilised genotype-specific HPV off-the-shelf probe designs from the ACD Catalog that have been successfully used in other publications. Therefore, the identification and use of HPV genotype-specific positive tissue as an additional positive control were not performed in this experiment since the use of external control tissues is often limited to novel probe designs, and only internal positive and negative controls were performed and assessed as recommended by the manufacturer to ensure accurate interpretation of the results based on mRNA integrity [8, 9]. This included running RNAscope for PPIB as a positive control to confirm functionality of the assay and DAPB as a negative control to determine any non-specific staining in the samples.

Whilst geographical and temporal factors could partially explain differences in prevalence between studies, the differences in prevalence seen in Australian cohorts indicate that specificity or sensitivity issues may be a more likely explanation [10].

In summary, both sensitivity and specificity are important aspects when making methodological decisions. We believe that future studies should prioritise prospective longitudinal designs, standardised HPV detection protocols – especially regarding strict anti-cross-contamination procedures - to resolve this controversy [1, 10].

## Data Availability

The studies mentioned to support the findings in this manuscript are available on MEDLINE.
